# High-Resolution Mapping of Photocatalytic Activity by Diffusion-Based and Tunneling Modes of Photo-Scanning Electrochemical Microscopy

**DOI:** 10.1021/acsnano.4c13276

**Published:** 2025-01-10

**Authors:** Tianyu Bo, Debjit Ghoshal, Logan M. Wilder, Elisa M. Miller, Michael V. Mirkin

**Affiliations:** † Department of Chemistry and Biochemistry, Queens College, Flushing, New York 11367, United States; § The Graduate Center of CUNY, New York, New York 10016, United States; ‡ Materials, Chemistry, and Computational Science Directorate, National Renewable Energy Laboratory, Golden, Colorado 80401, United States; ∥ Advanced Science Research Center at The Graduate Center, CUNY, New York, New York 10031, United States

**Keywords:** photoelectrocatalysis, scanning electrochemical microscopy, nanoelectrochemistry, tunneling, molybdenum disulfide, photoelectrochemistry, reactivity mapping

## Abstract

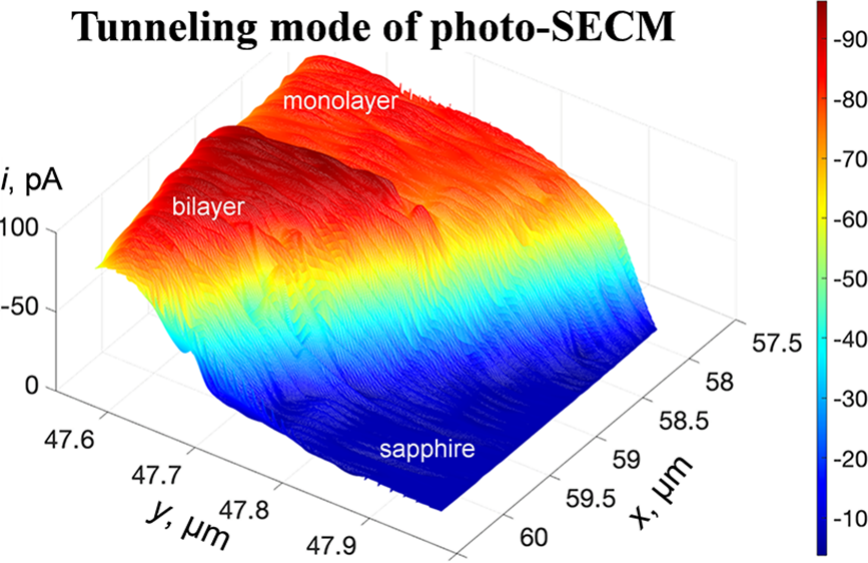

Semiconductor nanomaterials and nanostructured interfaces have important technological applications, ranging from fuel production to electrosynthesis. Their photocatalytic activity is known to be highly heterogeneous, both in an ensemble of nanomaterials and within a single entity. Photoelectrochemical imaging techniques are potentially useful for high-resolution mapping of photo(electro)catalytic active sites; however, the nanoscale spatial resolution required for such experiments has not yet been attained. In this article, we report photoreactivity imaging of two-dimensional MoS_2_ photocatalysts by two modes of photoscanning electrochemical microscopy (photo-SECM): diffusion and tunneling-based modes. Diffusion-based (feedback mode) photo-SECM is used to map the electron transfer and hydrogen evolution rates on mixed-phase MoS_2_ nanosheets and MoS_2_ chemical vapor deposition (CVD)-grown triangles. An extremely high resolution of photoelectrochemical imaging (about 1–2 nm) by the tunneling mode of the photo-SECM is demonstrated.

The activity of electrocatalysts and photo(electro)catalysts, which are widely employed in energy conversion and synthesis applications, is largely determined by microscopic surface sites, such as various defects, steps, facet edges, and corners. The kinetic heterogeneity of electrocatalytic surfaces has been extensively studied over the past few decades.^1−4^ More recently, heterogeneous photo(electro)catalytic activity has been investigated in different types of photoelectrodes, including faceted semiconductor microcrystals,^5−10^ photocatalysts/cocatalyst systems,^11−14^ and two-dimensional (2D) photocatalysts,^15−18^ such as MoS_2_ and other transition metal dichalcogenides (TMDCs). Most of the reported spatially resolved studies of photo(electro)catalytic surfaces employed either optical techniques, such as single-molecule fluorescence microscopy^8,19^ and transient absorption microscopy,^20^ or scanning probe microscopies, including conductive atomic force microscopy (AFM),^21,22^ potential-sensing AFM,^23,24^ and surface photovoltage Kelvin probe force microscopy (SPV-KPFM).^25−27^

Localized experiments employing electrochemical scanning probe microscopies can directly probe the rates of photo(electro)catalytic processes and have been gaining in popularity in the last two decades, especially for nanomaterials like 2D TMDCs.^28,29^ One insightful electrochemical scanning probe microscopy technique for TMDCs is scanning electrochemical cell microscopy (SECCM). SECCM has mapped the heterogeneous surface reactivity of mechanically exfoliated p-type WSe_2_ nanosheets and observed lower photoelectrochemical rates for outer-sphere redox couples at step/edge defects.^16^ A somewhat similar approach—microdroplet-based photoelectrochemical microscopy—showed that edge and defect sites acting as charge carrier recombination centers are predominantly responsible for the decreased photoelectrochemical activity of mechanically exfoliated nanosheets in both WSe_2_ and MoSe_2_.^30^ Scanning photocurrent microscopy mapping of individual MoSe_2_ nanoflakes also showed that charge carrier recombination is greater near perimeter edges.^15,17^ By contrast, the nanosheet’s edges and engineered defects exhibit significantly enhanced activity toward hydrogen evolution reaction (HER),^16,31^ and CO_2_ reduction,^32^ suggesting that edge sites serve as both catalytic sites for HER and detrimental charge recombination centers. In other microscopy experiments on TMDCs, scanning photoelectrochemical microscopy revealed the presence of n- and p-type domains within the same exfoliated MoS_2_ nanoflake. The photocurrents from individual n- and p-type domains oppose each other, resulting in a lower overall current response of MoS_2_ flakes.^17^ While these studies have provided valuable information about the heterogeneity of electron transfer (ET) and photoelectrocatalytic surface activity of TMDC flakes, relatively low (i.e., micrometer or submicrometer-scale) spatial resolution of the employed techniques limited their capacities for identifying what caused the kinetic heterogeneity.

Scanning electrochemical microscopy (SECM) with nanometer-sized tips can attain a high spatial resolution (e.g., sub-20 nm^3,33^) and measure products and intermediates of the electrocatalytic reactions on the nanoscale. By contrast, most of the reported photo-SECM experiments employed micrometer-sized electrodes in studies of photoelectrochemical processes^34−43^ and for screening and optimization of photo(electro)catalysts.^44−48^ We have recently used photo-SECM equipped with a nanotip for spatially resolved studies of overall water splitting (OWS) at single Al-doped SrTiO_3_/Rh_2–*y*_Cr_*y*_O_3_^49^ and P-doped BiVO_4_^50^ microcrystals. Importantly, this technique is capable of measuring local fluxes of products of photocatalytic processes without applying an external bias to the sample, which is essential for probing the OWS and other photocatalytic processes. However, the 100–200 nm radius tips employed in our previous work (refs (49 and 50)) were too large for probing individual cocatalyst particles or active site mapping.

Here, we employ much smaller tips to map photo(electro)catalytic activities of mixed-phase MoS_2_ nanosheets and MoS_2_ chemical vapor deposition (CVD)-grown triangles with a few tens of nm resolution using feedback (i.e., diffusion-based) mode of SECM (Figure 1A,C-i). We also introduce a new tunneling mode of photo-SECM, which enables photoelectrochemical imaging with extremely high resolution on the order of 1–2 nm and obtain tunneling photo-SECM images of topography and surface reactivity based on ET (Figure 1B-i) and photoelectrochemical HER (Figure 1C-ii) at the MoS_2_ surface. In the latter case, a sharp transition from the negative SECM feedback (Figure 1C-i) to tunneling response (Figure 1C-ii) occurred under illumination, allowing us to investigate the differences in electrochemical tunneling at pseudometallic (1T′) MoS_2_ and semiconducting (2H) MoS_2_ surfaces and also between MoS_2_ monolayers and bilayers.

**Figure 1:**
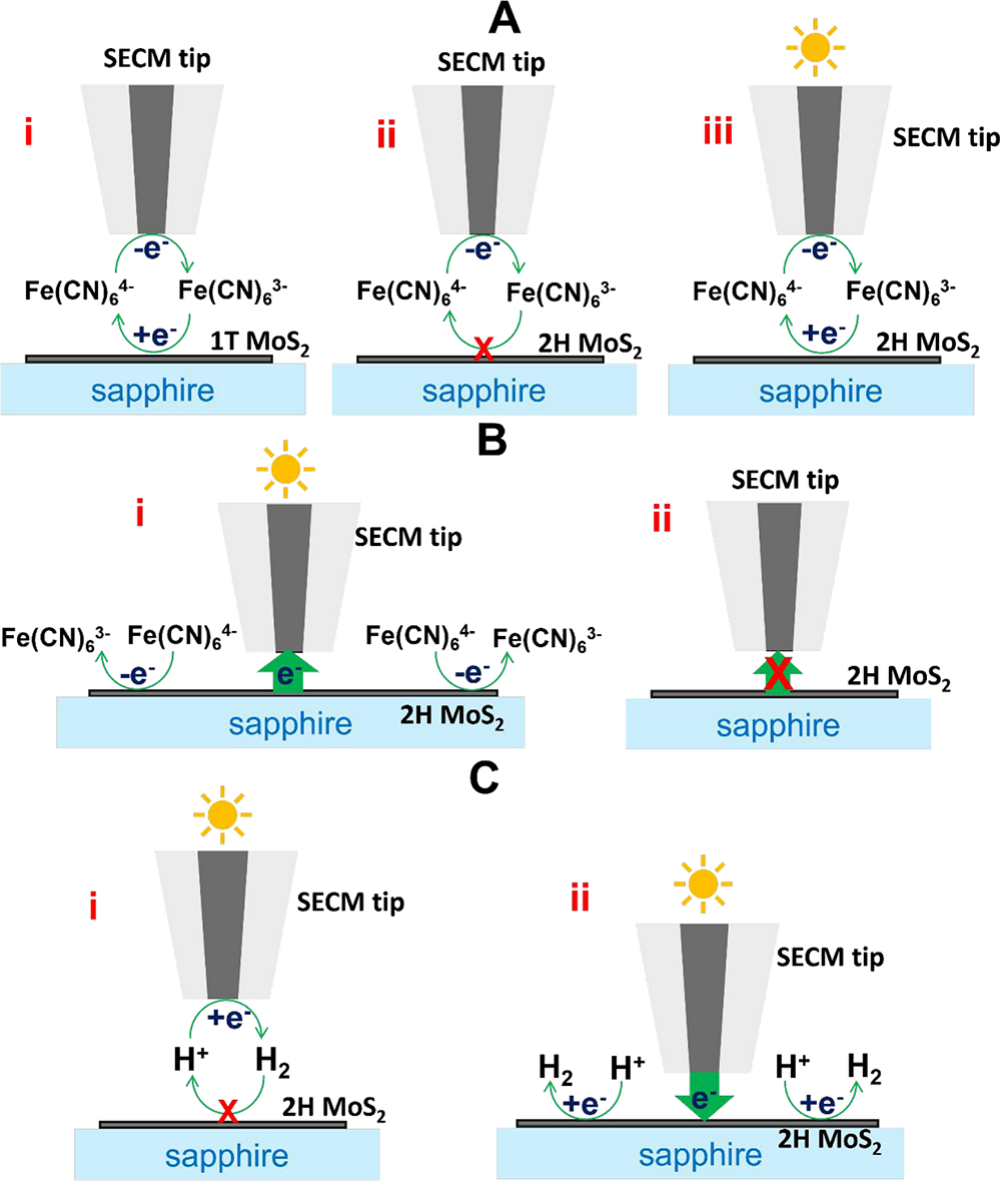
Schematic representation of diffusion-based and tunneling mode SECM experiments at semiconducting (2H) and pseudometallic (1T′) MoS_2_ nanomaterials in the dark and under illumination. (A) Diffusion-based, feedback mode experiments with a reversible redox mediator. Positive SECM feedback is due to mediator regeneration at 1T′ MoS_2_ (i). 2H MoS_2_ produces negative feedback in the dark (ii) and positive feedback under illumination (iii). (B) Electron tunneling between the Pt tip and 2H MoS_2_ occurs under illumination (i) but not in the dark (ii). (C) 2H MoS_2_ does not oxidize hydrogen (i; negative feedback) but reduces protons in tunneling mode (ii).

## Results and Discussion

### Diffusion-Based vs Tunneling Mode Overview

In the diffusion-based, feedback mode experiment (Figure 1A), a nanometer-sized SECM probe is brought within a short distance from the sample. The electrolyte contains either a reduced or oxidized form of a redox mediator (e.g., ferrocyanide; Fe(CN)_6_^4–^), and the tip potential (*E*_T_) is such that the mediator oxidation occurs at a rate governed by diffusion. When the separation distance between the tip and substrate (*d*) is small enough (i.e., comparable to tip radius, *a*), Fe(CN)_6_^3–^—the product of the oxidation occurring at the tip surface—can be reduced at the substrate. If a mixed-phase MoS_2_ nanosheet (comprising 2H and 1T′ regions) is used as a sample, the rapid regeneration of the mediator occurs in the dark when the tip approaches a 1T′ portion of the flake (Figure 1A-i), and the tip current increases with decreasing *d* (positive feedback; the tip current near the surface is higher than its value in the bulk solution; *i*_T_ > *i*_T,∞_). This experiment can be done either with a negative potential applied to a mixed-phase MoS_2_ nanosheet immobilized on a conductive support or with an unbiased nanosheet placed on an insulating surface (e.g., sapphire). In the latter case, the potential of the 1T′ MoS_2_ surface is significantly more negative than the standard potential of the Fe(CN)_6_^3/4–^ couple because the solution contains only ferrocyanide species. In contrast, the mediator regeneration is slow in the dark when the tip approaches the 2H MoS_2_ phase (Figure 1A-ii), and *i*_T_ decreases with decreasing *d* because of the hindered diffusion of the mediator to its surface (negative feedback; *i*_T_ < *i*_T,∞_). However, positive feedback can be observed at the 2H MoS_2_ surface under broadband range UV–vis illumination because of fast Fe(CN)_6_^3–^ reduction (Figure 1A-iii), whereas the illumination does not impact the 1T′ MoS_2_ mediator regeneration.

Bringing an SECM tip very close (*d* < 3 nm^51^) to the surface of a conductive sample causes the transition from the feedback response to electron tunneling between the tip and the substrate. Unlike STM tunneling, where voltage is applied between the tip and substrate, in electrochemical tunneling experiments the sample is attached to an insulating support, and the current is produced by electron tunneling between the tip and the conductive sample along with electrooxidation (or reduction) of the redox mediator at the substrate surface (Figure 1B-i,C-ii). At a very short separation distance, a conductive specimen acts as a part of the nanotip so that voltammograms can be obtained without attaching it to the electrode surface, which we have previously demonstrated with metal nanoparticles.^51^ Tunneling SECM experiments can also be performed at flat samples, such as TMDC flakes,^52^ but no imaging of such surfaces has yet been reported. By eliminating the diffusion of electroactive species in the tip/substrate gap, which limits the attainable lateral resolution of conventional SECM imaging, the tunneling mode of SECM is expected to produce high-resolution surface maps.^53^

### Feedback Mode SECM and Photo-SECM Mapping of Mixed Phase MoS_2_ Nanosheets

**Figure 2:**
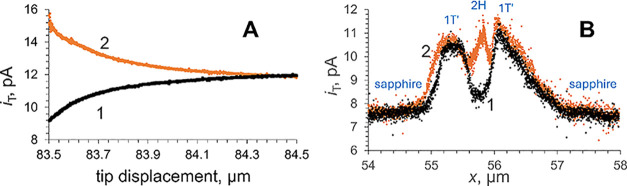
Mapping dark reactivity and photoreactivity of a mixed-phase MoS_2_ nanosheet by the feedback mode of SECM. (A) Approach curves and (B) lateral scans obtained with the same carbon nanotip in the dark (1) and under illumination (2). *a* = 42 nm. Solution contained 1 mM K_4_Fe(CN)_6_ in 0.1 M KCl. *E*_T_ = 0.4 V vs Ag/AgCl.

In the first set of experiments, we used the feedback mode of SECM to image mixed-phase MoS_2_ nanosheets chemically exfoliated from bulk MoS_2_ by using *n*-butyl lithium. These nanosheets (a few nm thick and 100s of nanometers laterally, Figure S2) contain patches of 2H MoS_2_ surrounded by 1T′ MoS_2_.^33,52^ Two current versus distance (*i*_T_–*d*) curves in Figure 2A were obtained with the same 42 nm-radius carbon nanotip approaching the 2H portion of the MoS_2_ nanosheet in the dark (curve 1) and under illumination (curve 2) in a solution containing 1 mM ferrocyanide. The negative feedback response in curve 1 and positive feedback in curve 2 suggest that ferricyanide reduction occurs at 2H MoS_2_ under illumination but not in the dark (cf. Figure 1A-ii,iii). When the same tip was scanned laterally (i.e., in the *x*–*y* plane) ∼30 nm above a mixed-phase MoS_2_ nanosheet immobilized on the sapphire support, negative feedback was observed over three segments of the line scan obtained in the dark (curve 1 in Figure 2B), while positive feedback could be seen over two other segments. While positive SECM feedback could only be measured over the conductive 1T′ MoS_2_ portion of the surface,^32^ negative feedback could be produced either by the insulating sapphire or by the 2H MoS_2_ portion of the surface, which does not regenerate ferrocyanide mediator in the dark.

The line scan of the same portion of the mixed-phase MoS_2_ nanosheet obtained with the same tip under illumination (Figure 2B, curve 2) overlaps with the scan recorded in the dark (curve 1) over the sapphire surface, where the magnitude of the negative SECM feedback is independent of light. By contrast, when the tip is over the 2H MoS_2_ portion of the nanosheet, the response changes from negative to positive feedback when the sample is illuminated. A minor *i*_T_ increase over the 1T′ MoS_2_ portions is probably due to the neighboring 2H regions.

As discussed previously, the spatial resolution of photoreactivity mapping in Figure 2B is determined not by the size of the light spot on the sample surface (which is of the order of a few micrometers^62^), but by the tip radius, *a* = 42 nm. A much higher spatial resolution can be obtained in tunneling SECM experiments by eliminating the diffusion in the tip/substrate gap.^53^

### Tunneling Mode of Photo-SECM

**Figure 3:**
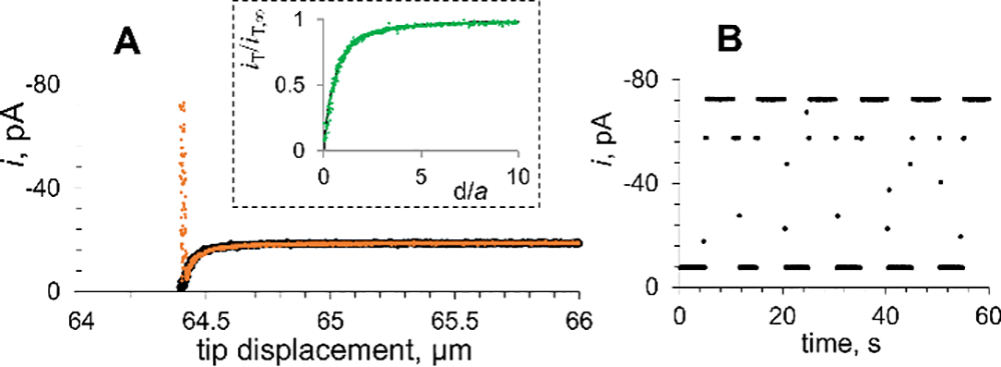
HER-based approach curves and chopped-light transients obtained over a MoS_2_ CVD-grown triangle attached to the sapphire surface. (A) Approach curves obtained in the dark (black curve) and under illumination (orange curve) with the same *a* = 59 nm Pt tip approaching the same spot on the MoS_2_ surface. Inset: a portion of the experimental approach curve recorded without illumination (green symbols) fitted to the theory for the pure negative feedback (black solid line;^54^ RG = 2). (B) Tunneling mode chopped-light current transients recorded with the same tip touching the sample surface. Solution contained 1 mM HClO_4_ in 0.1 M KCl. *E*_T_ = −0.7 V vs Ag/AgCl.

The tunneling mode of SECM was used to probe both mixed-phase MoS_2_ nanosheets and 2H MoS_2_ CVD-grown triangles. With a MoS_2_ CVD-grown triangle, the difference between the tunneling SECM response in the dark and under broadband range UV–vis illumination is most striking for HER (Figure 1B-ii,C-ii). The transition from diffusion-based SECM feedback to tunneling can be seen by comparing the approach curves obtained in the dark (Figure 3A, black curve) and under illumination (Figure 3A, orange curve). In the dark, *i*_T_ decreases monotonically as the tip approaches a MoS_2_ CVD-grown triangle, and the current-distance curve fits the theory for pure negative feedback (the inset in Figure 3A). The approach curve under illumination (Figure 3A, orange curve) is indistinguishable from that obtained in the dark (black curve) until the onset of the tunneling current occurs at a few nanometers of separation distance. The magnitude of the photocurrent produced by the HER at the illuminated MoS_2_ CVD-grown triangle can be evaluated from the chopped-light transients (Figure 3B). The *i*_T_ transients are reproducible and very fast with a rise time in the range of milliseconds.

**Figure 4:**
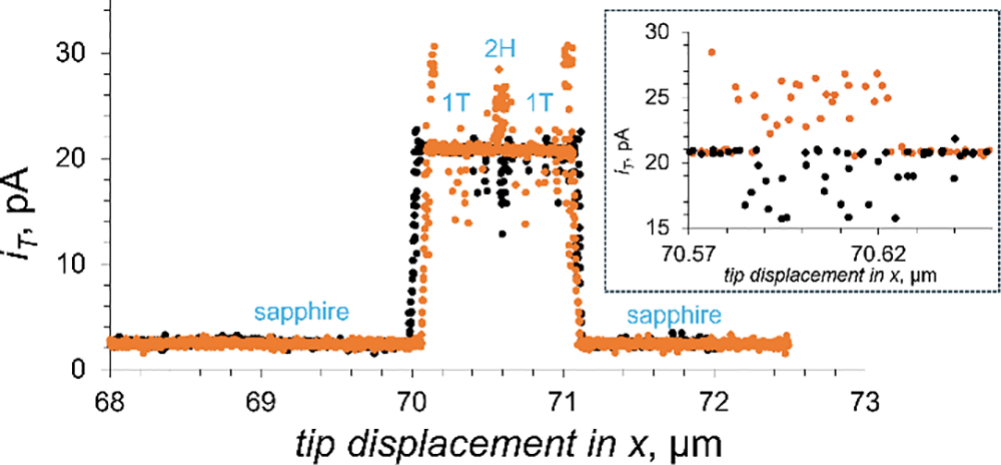
Lateral scans in tunneling mode recorded over a mixed-phase MoS_2_ nanosheet in 1 mM Fe(CN)_6_^4–^ in 0.1 M KCl in the dark (black circles) and under illumination (orange circles). *E*_T_ = 0.4 V vs Ag/AgCl. Carbon tip, *a* = 45 nm. The inset shows the magnification of the 2H region.

An example of tunneling mode SECM reactivity mapping of a mixed-phase MoS_2_ nanosheet is shown in Figure 4. The black and orange curves represent two line scans obtained with the same carbon tip over the same mixed-phase MoS_2_ nanosheet in the dark and under broad-band range UV–vis illumination, respectively. Similar to line scans recorded in feedback mode over a mixed-phase MoS_2_ nanosheet (Figure 2B), the tunneling current over the sapphire surface is very low both in the absence and presence of light, while the current recorded over 1T′ regions is much higher and essentially light-independent, and current over 2H MoS_2_ is much higher under illumination than in the dark. However, there are also major differences: the current changes corresponding to transitions between different surfaces are much sharper in tunneling mode line scans (Figure 4) than in the corresponding feedback mode curves (Figure 2B), and the magnitudes of current changes are significantly larger. These differences point to a much higher lateral resolution of the tunneling mode reactivity mapping, which enabled the visualization of a narrow (∼50 nm) segment corresponding to a small patch of 2H MoS_2_ surface within the nanosheet, which would be hard to observe with a lower resolution feedback-mode photo-SECM.

**Figure 5:**
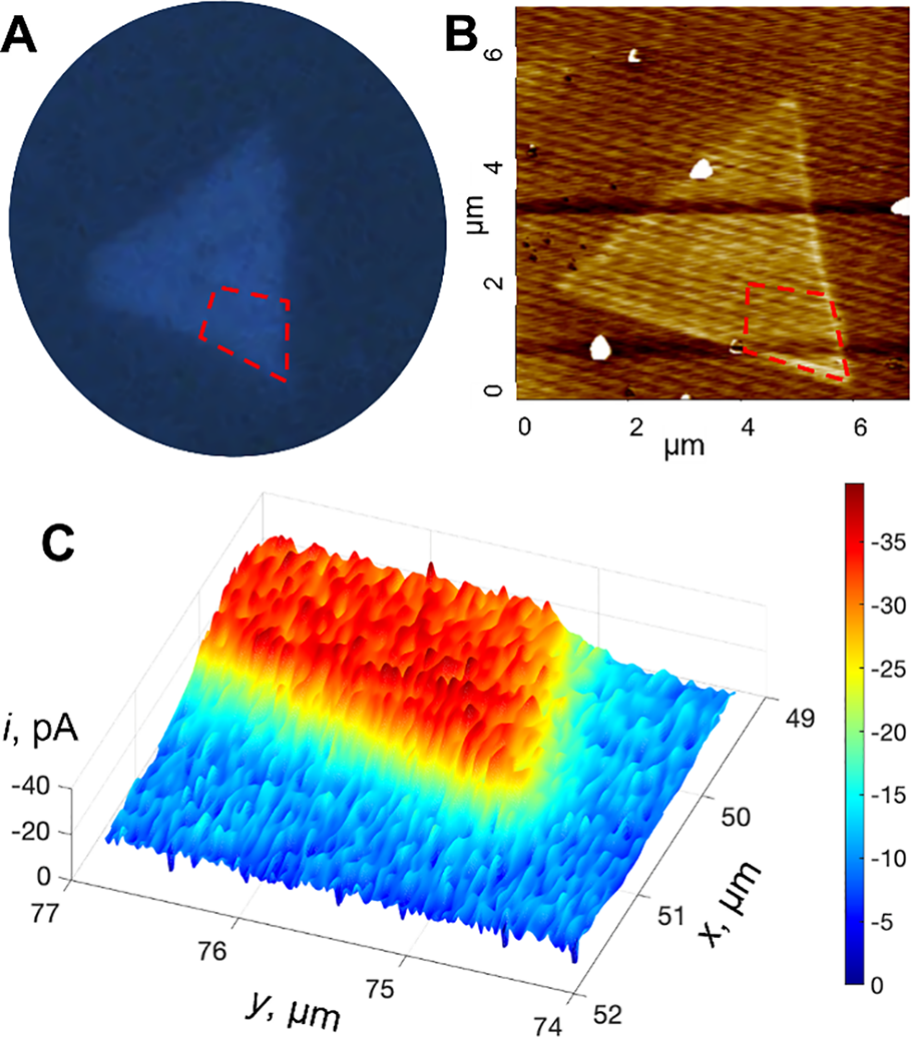
Imaging a MoS_2_ monolayer triangle on a Formvar-coated carbon TEM grid. The dashed polygons in (A) optical and (B) AFM images show a portion of the triangle mapped by the (C) tunneling mode of photo-SECM under UV–vis illumination with the tip current produced by HER. Solution contained 1 mM HClO_4_ in 0.1 M KCl. *E*_T_ = −0.7 V vs Ag/AgCl. *a* = 60 nm.

Tunneling SECM photoreactivity mapping of HER on a MoS_2_ CVD-grown monolayer triangle is shown in Figure 5. A dashed line polygon in the optical micrograph (Figure 5A) and the corresponding AFM image (Figure 5B) shows approximately the area of the triangle mapped with a 60 nm Pt SECM tip (Figure 5C); the correlation between AFM (B) and SECM (C) images is not exact because of two different experimental setups used. Except for a sharp transition between the inert Formvar surface and the photocatalytically active MoS_2_ monolayer triangle, the photoreactivity map is essentially uniform, showing neither the domain structure reported for exfoliated multilayer flakes^17^ nor decrease in the current near perimeter edges observed for multilayer MoSe_2_ flakes.^15^ The edges also do not exhibit increased catalytic activity for HER measured by SECM at exfoliated 2H MoS_2_ flakes in the dark.^33^

### Ultrahigh-Resolution Mapping of MoS_2_ CVD-Grown Triangles by Tunneling Photo-SECM

**Figure 6:**
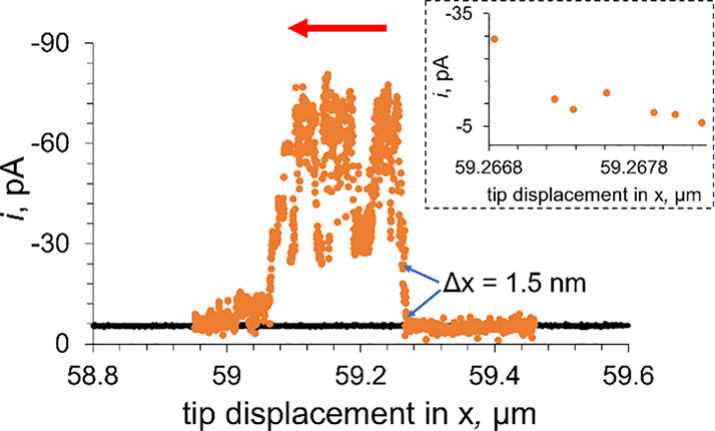
Tunneling mode lateral scans over a MoS_2_ monolayer triangle on the sapphire support recorded in the absence of light (black curve) and under illumination (orange) in 1 mM HClO_4_ in 0.1 M KCl. The red arrow indicates the scan direction. Inset: magnified portion of the line scan corresponding to the 1.5 nm tip displacement over the edge of the triangle indicated by blue arrows. *E*_T_ = −0.7 V vs Ag/AgCl, *a* = 60 nm.

To evaluate the lateral resolution of tunneling mode photo-SECM imaging, two line scans over the edge of a MoS_2_ CVD-grown monolayer triangle in tunneling SECM mode were recorded in the dark (Figure 6, black curve) and under broadband range UV–vis illumination (Figure 6, orange curve). In the absence of light, the MoS_2_ monolayer triangle is electrochemically inert, the line scan is featureless, and the tip current is much lower than that of its bulk (*i*_T,∞_ = 19 pA) because of a very small separation distance. The tip current recorded over sapphire was very similar under illumination and in the dark. However, the current drastically increases when the Pt tip is moved across the edge of the irradiated 2H MoS_2_ surface. Importantly, the tip displacement corresponding to this change is extremely small, ca. 1–2 nm. Moreover, the current increased essentially monotonically within the 1.5 nm displacement (inset in Figure 6). This observation suggests that photoelectrochemical imaging in the tunneling mode of SECM can offer extremely high lateral resolution.

**Figure 7:**
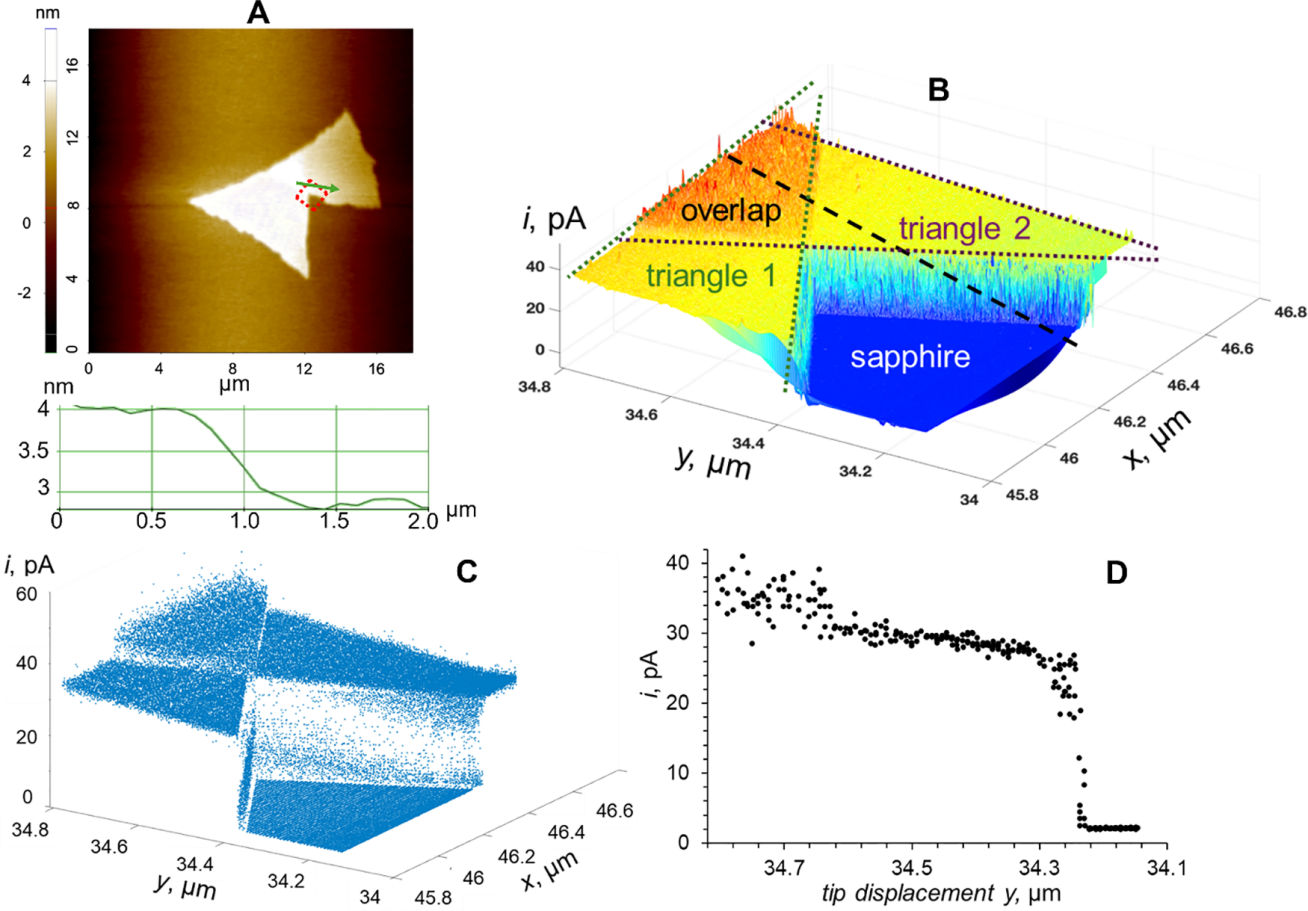
Correlated AFM and tunneling SECM images of partially overlapping MoS_2_ CVD-grown triangles on the sapphire support. (A) AFM image of the triangles with the dotted square approximately showing the area mapped by photo-SECM. The green arrow indicates the scan direction in the corresponding cross section. (B) Colored 3D tunneling photo-SECM image with green and purple dotted lines showing the areas of two triangles and the orange region corresponding to their overlap. (C) Same data as in panel B in the form of the raw pixelated image. (D) Tunneling photo-SECM scan along the specified trajectory (dashed black line in panel B) shows the transitions from the bare support to the single MoS_2_ layer (*y* ≈ 34.23 μm) and then to the bilayer (*y* ≈ 34.63 μm). The current was due to the oxidation of 1 mM Fc in 0.1 M KCl at the 45 nm Pt tip. *E*_T_ = 0.4 V vs Ag/AgCl.

The high spatial resolution and sensitivity of the tunneling mode of photo-SECM mapping are evident in the image of two overlapping MoS_2_ CVD-grown triangles (Figure 7) in which the current was due to the oxidation of ferrocyanide. An AFM image (Figure 7A) shows two partially overlapping triangles on the sapphire support and the area mapped by SECM located within the red dotted square. The cross-section represents the line scan from the bilayer (i.e., the overlap region) to the single MoS_2_ layer, as shown by the green arrow. Green and purple dotted lines in Figure 7B designate the areas of both triangles in the colored 3D (MATLAB graphics) tunneling photo-SECM image in which the orange region corresponds to the overlap of these triangles. The black dashed line in Figure 7B approximately represents the trajectory of the photo-SECM line scan shown in Figure 7D. Figure 7C shows the same data as in Figure 7B in the form of the raw pixelated image, exhibiting a sharp transition between the inert sapphire and MoS_2_ monolayer triangle surface and a smaller but well-defined transition from a monolayer to a bilayer region. Very small (a few nanometers) tip displacements corresponding to these transitions can be seen in the line scan (Figure 7D).

One striking feature in Figure 7C is the surprisingly stable *i*_T_ with a relatively low noise recorded over all regions (i.e., sapphire surface, both monolayer triangles, and a bilayer region). Such a stable tunneling current is only possible if the tip/sample separation distance is constant on the sub-nm scale. The SECM image and line scan in Figure 7 were obtained by scanning the tip laterally in the *x*–*y* plane over the sample without any control of *d*. Because of the inevitably imperfect tip alignment with respect to the substrate surface and slow thermal drift in the piezo, maintaining strictly constant *d* for the entire relatively large SECM image (several hundred nm^2^) is not feasible. A plausible explanation of the high current stability is that the nanoshaft of the glass-sealed tip bends when the tip touches the substrate and slightly presses against the sample surface, maintaining physical contact between a small conductive feature on the tip surface and the sample, thus forming a stable tunneling junction. Without such stabilization, the tip oscillations produce a high-amplitude noise (see, e.g., Figure 6) noticed in previous nano-SECM studies.^52,55^

Although the tunneling current in Figure 7 is due to the diffusion of ferrocene methanol (Fc) to and its photooxidation on the MoS_2_ surface, the recorded *i*_T_ is ∼1–2 orders of magnitude lower than the expected nA-range steady-state diffusion-limited current of 1 mM Fc to an electrode shaped as a unilateral triangle with the 10 μm side length. This observation is different from the tunneling SECM data obtained with metal nanoparticles, where the measured *i*_T_ was close to the diffusion-limited current of the redox mediator to a nanoparticle,^51^ and from tunneling experiments at more conductive nanoflakes (e.g., MXenes) that yielded nA-range tip current values.^52^ The much lower tip currents measured here are limited either by the tip/MoS_2_ contact resistance or by the transport of holes to the tip in the MoS_2_ layer. The former assumption is not plausible since the tips made of different materials (i.e., Pt in Figures 5C and 7B–D, and carbon in Figure 4) produced comparable tunneling photocurrents. The same conclusion can be drawn from tunneling steady-state voltammograms of Fe(CN)_6_^4–^ oxidation at a ∼46 nm-radius carbon tip (Figure S1A in the Supporting Information) and a 34 nm Pt tip (Figure S1B) touching an illuminated MoS_2_ CVD-grown triangle. In both cases, the halfwave potentials are very close to the standard potentials of Fc and ferrocyanide, respectively, indicating that the tip/sample contact resistance is relatively small. The measured tip current is apparently limited by carrier transport, which could be faster in an MoS_2_ bilayer than in a monolayer due to a difference in sulfur vacancies.^56^

**Figure 8:**
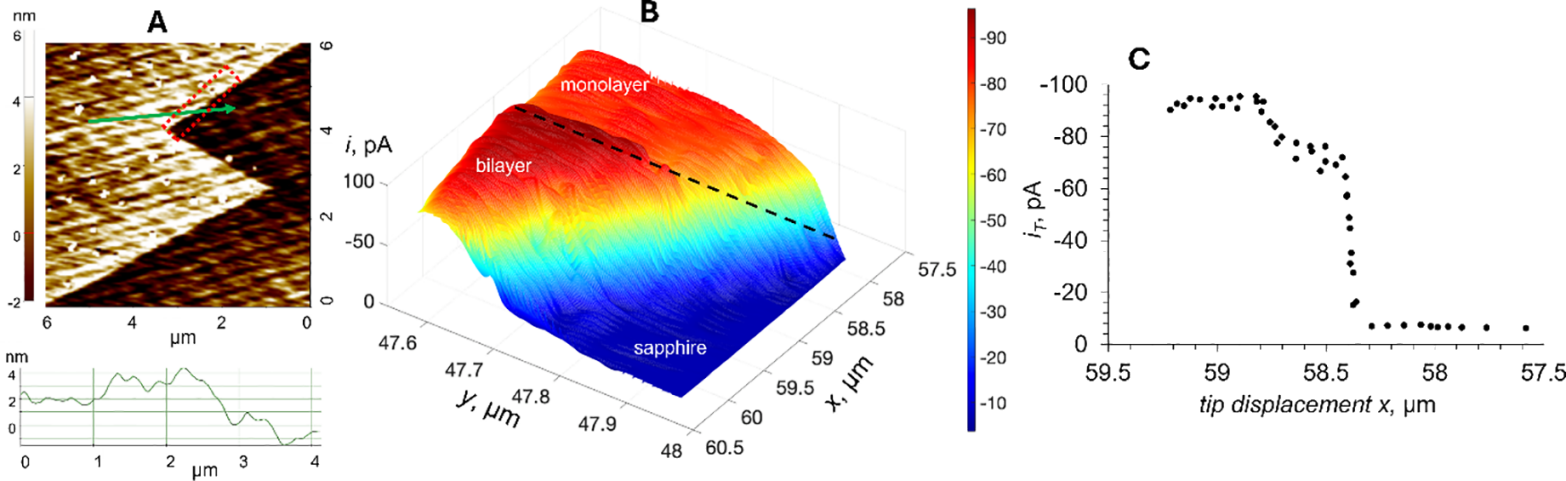
AFM (A) and tunneling photo-SECM (B) images of two partially overlapping MoS_2_ CVD-grown monolayer triangles. (A) Dotted rectangle in the AFM image shows approximately the area mapped by photo-SECM, and the green arrow indicates the scan direction in the corresponding cross section. (B) Tunneling photo-SECM image obtained under illumination in a 1 mM HClO_4_ solution in 0.1 M KCl with a 60 nm Pt tip. *E*_T_ = −0.7 V versus Ag/AgCl. (C) Lateral line scan along the specified trajectory (dashed black line in panel B).

High-resolution reactivity mapping by tunneling SECM can be done for different ET and photocatalytic processes. A tunneling photo-SECM image of two overlapping MoS_2_ triangles on a sapphire support was obtained by using HER as the source of the tip current (Figure 8). The dashed red rectangle in the AFM image of these triangles (Figure 8A) shows approximately the area mapped by SECM (Figure 8B), and the green line corresponds to the cross-section.

The three segments discernible in this cross-section represent the sapphire surface and two partially overlapping triangles. There are two corresponding current steps in the SECM image (Figure 8B), a larger change for the boundary between the sapphire and a MoS_2_ triangle and a smaller one representing the monolayer/bilayer boundary. The lateral line scan (Figure 8C) along the dashed black line in panel B shows the transitions from the sapphire surface to the single MoS_2_ layer (at 58.42 μm) and then to the bilayer (at 58.75 μm).

## Conclusions

In conclusion, we demonstrated high-resolution mapping of surface reactivity by two modes of photo-SECM. The diffusion-based, feedback mode of photo-SECM, which was previously employed for single-particle measurements with relatively large tips, is suitable for mapping photoreactivity with the resolution of a few tens of nanometers. A much higher (i.e., a few nm range) resolution was attained using the tunneling mode of photo-SECM, in which the source of the tip current was either ET involving a dissolved redox species or photoelectrocatalytic HER at the illuminated 2H MoS_2_ surface. This technique is sufficiently sensitive to differentiate between a single-layer MoS_2_ triangle and a bilayer formed by two overlapping triangles based on the differences in the lateral charge transfer rate. Further experiments and simulations are required to better understand the underlying mechanisms and extract quantitative information from tunneling photo-SECM measurements.

## Methods and Experimental Section

### Chemicals and Materials

Potassium ferrocyanide (99%), HClO_4_ (70%), and NaClO_4_ (99%) were purchased from Sigma-Aldrich and used as received. Aqueous solutions were prepared using deionized water from a Milli-Q Advantage A10 system equipped with a Q-Gard T2 Pak, a quantum TEX cartridge, and a VOC Pak.

Chemically exfoliated MoS_2_ was generated as follows: MoS_2_ (500 mg, Millipore Sigma) was heated to 150 °C for 1 h in ambient air to remove residual water and then brought into a N_2_ glovebox. A solution of *n*-butyllithium in hexane (10 mL, 1.6 M, Millipore Sigma) was added to the MoS_2_, and the resulting mixture was stirred for 72 h. Next, while in the N_2_ glovebox, the exfoliated MoS_2_ was filtered over a poly(ether-sulfone) 0.2 μm filter (ThermoFisher Scientific) and washed with 20 mL of hexane, and the washing step is repeated 5 times. The filter assembly was removed from the N_2_ glovebox. (A safety precaution must be noted here, as residual *n*-butyllithium may catch fire when exposed to ambient air.) Next, DI water (200 mL) was used to resuspend the MoS_2_ exfoliated flakes. (An additional safety precaution must be noted here, as residual *n*-butyllithium may catch fire when exposed to water.) The MoS_2_ in water was cooled with an ice bath, and the tip was sonicated for 1 h. Unexfoliated MoS_2_ was removed from the solution by centrifuging for 30 min at 2 kg, followed by discarding the precipitate and keeping the supernatant. The exfoliated MoS_2_ solution was stored in the dark at 6 ± 2 °C. This protocol results in mixed-phase (1T′/2H) MoS_2_ nanosheets (Figure S2). Two Raman spectra of the mixed-phase exfoliated MoS_2_ on a Si substrate are shown in Figure S5. As expected, mixed 1T′ and 2H characters are apparent from the spectra, with different areas of the sample showing different levels of 1T′/2H ratio (qualitative). In the spectrum labeled Area 1, peaks at 155, 232, and 331 cm^–1^ result from the *J*_1_, *J*_2_, and *J*_3_ active modes of the 1T′ phase, which are very weak in the Area 2 spectrum. The peaks at 378.5 and 402.9 cm^–1^ result from the E^1^_2g_ and A_1g_ modes of the 2H phase and are much stronger in Area 2 than Area 1.^57,58^

2H MoS_2_ monolayer triangles (Figure S3) were synthesized by CVD in a 3-zone 2-inch tube furnace with a separate heating belt for heating sulfur (S). Double-side polished C-plane sapphire wafers <0001> off *M*-plane <1–100> 0.2 ± 0.1° from University Wafers were used as substrates for growth. The sapphire wafer was sequentially sonicated in acetone, ethanol, and finally in isopropyl alcohol (IPA). The cleaned sapphire wafers were loaded in the CVD furnace along with the solid phase precursors (S powder, Sigma-Aldrich 99.97%; and MoO_3_ powder, Sigma-Aldrich 99.998%) for MoS_2_ growth. The substrate was loaded in zone 3 of the furnace downstream from the precursors. MoO_3_ powder was loaded in zone 1 of the furnace. S was loaded most upstream outside the heating zone of the tube furnace and was heated with an external heating belt. All the 3 zones of the CVD furnace were maintained at 800 °C during growth. The quartz tube had a flow of 50 sccm of Ar at 625 Torr. The S was sublimated at 180 °C once zone 3 of the furnace reached 650 °C for 20 min. Post 20 min, the furnace was opened, and the system was allowed to cool in Ar flow of 50 sccm. After growth, most of the substrate surface is covered by monolayer MoS_2_ triangles. In certain areas, the overlapping growth of two triangles creates bilayer regions. Unlike bilayers formed by stacking two monolayers via transfer processes (wet or dry), these bilayer regions form naturally during growth, eliminating the likelihood of trapped air bubbles between layers. The MoS_2_ triangles on sapphire were removed from the furnace and stored in a N_2_ box until needed.

### Fabrication and Characterization of SECM Tips

Platinum nanoelectrodes were prepared by pulling and heat sealing 25 μm diameter Pt wires (Goodfellow) into borosilicate glass capillaries (Drummond; OD—1.0 mm; ID—0.2 mm) under vacuum with a P-2000 laser pipet puller from Sutter Instruments, polished on a 50 nm alumina pad (Precision Surfaces International) under video microscopic control as described previously^55^ and sonicated in deionized water for 1 min. A microforge (model MF-900, Narishige, Tokyo, Japan) was used to reduce the RG (i.e., the ratio of the glass radius to that of the conductive tip) of the tapered tip. The nanoelectrodes were characterized by steady-state voltammetry, SECM approach curves, and transmission electron microscopy (TEM) (Figure S4).

Carbon nanotips were prepared by CVD of carbon inside prepulled quartz nanopipettes, as described previously.^59,60^ Briefly, nanopipettes were produced by pulling quartz capillaries (1.0 mm o.d., 0.5 mm i.d.; Sutter Instruments) with a laser pipet puller (P-2000, Sutter Instruments). Carbon was deposited onto the inner pipet wall by CVD at 950 °C, using methane as a carbon source and argon as a protector (argon/methane: 1/1). A 1 h deposition time was sufficient to fill the nanopipettes nearly completely with carbon. The electrostatic discharge (ESD) protection was used during all steps of the electrode preparation to prevent nanometer-scale damage to the tip.^61^ The *a* value was validated, and the electrode geometry was checked by TEM, using a JEOL JEM-1400 instrument (HV = 120 kV).

Nanoelectrodes were tested by steady-state voltammetry of 1 mM ferrocyanide. Voltammograms were obtained with a CHI-760E electrochemical workstation (CH Instruments). The three-electrode setup was used with an Ag/AgCl reference electrode with a porous Teflon tip (CH Instruments) and a 1 mm Pt wire as a counter electrode.

### Sample Characterization by AFM

An XE-120 scanning probe microscope (Park Systems) was used for imaging the samples. Topography imaging was carried out in noncontact mode using PPP-NCHR ADM probes (Nanosensors)

### SECM Procedures

SECM experiments were carried out using a home-built instrument similar to that described previously.^33^ The SECM was mounted on an AVI-200S active vibration isolation platform placed inside a NanoVault acoustic enclosure (Herzan). The actuation for imaging and fine positioning was through a P-621 PI Hera 3D nanopositioning stage (Physik Instrumente) driven by an E-725.3CDA multiaxis piezo controller (Physik Instrumente) and controlled by home-written LabView software. One mM ferrocyanide was used as a redox mediator in feedback and tunneling mode SECM experiments with a tip potential, *E*_T_ = 0.4 V vs Ag/AgCl, corresponding to diffusion-limited oxidation current, while the substrate was unbiased. For HER measurements, the solution was replaced with 1 mM HClO_4_, and *E*_T_ was changed to −0.7 V vs Ag/AgCl. To obtain an approach curve, the tip was first brought within a ∼20 μm vertical distance from the substrate using a manual micromanipulator. Then, the tip was moved toward the substrate using the vertical Z piezo stage with a relatively high approach velocity (e.g., 0.1 μm/s), which was changed to 0.05 and 0.01 μm/s within 5 and 1 μm away from the substrate, respectively. Eventually, the approaching velocity reached a much slower value, e.g., ∼1–5 nm/s within a distance of 10 nm from the substrate. To locate a desired sample, the SECM tip was brought within 1–2 tip radii from the substrate surface as described previously and then scanned laterally in the *x*–*y* plane above it.^3,33^ Both the 2H phase of mixed-phase MoS_2_ nanosheets and 2H MoS_2_ CVD-grown triangles showed negative feedback in the dark, while the 1T′ phase showed positive feedback either in the dark or under illumination. Tunneling mode images were obtained by positioning an SECM tip above the sample surface within a tunneling region (∼2–3 nm) and then moving the tip laterally in both *X* and *Y* directions within a preset scanning area. All experiments were performed in a Faraday cage at room temperature (23 ± 2 °C).

Photo-SECM experiments were carried out using a home-built setup for through-tip illumination of the sample like that described previously.^62,63^ Briefly, for the broad-band UV–vis illumination, an optical setup (Newport Corporation) consisted of an OPS-A500 500 W power supply, a 250 W HgXe lamp with a fiber bundle focusing assembly (model 77776) attached to its housing (model 67005), and a broad wavelength range optical fiber (model 78277, UV–vis Single Fiber Cable) with a core diameter of 1 mm. An IR cutoff FSQ-KG3 glass filter (Newport) was used to minimize sample heating during the experiment. In experiments with Pt tips, the electrode body served as a light guide,^62^ and an optical fiber was optically connected to it using a lens system (Thorlabs) that consists of a lens tube (SM05) installed with two plano-convexes with a focal length, *f* = 10 mm (ASL1210-UV).^63^ In this way, light from the optical fiber was collimated by the first lens and focused by the second lens to a light spot of ∼1 mm at the back of the tip, while the glass sheath of the nanoelectrode acted as a light-guide, focalizing the radiation on the substrate area facing the tip. In experiments with C tips, this setup had to be modified because of the strong light absorption by carbon—the light coming from the optical fiber was directed parallel to the glass wall of the tip.

## Supporting Information Available

Characterization of MoS_2_ mixed phase nanosheets and CVD-grown triangles, TEM images of Pt and C nanoelectrodes, and tunneling mode voltammograms (PDF)
