# Supplementary material for: High-Resolution Mapping of Photocatalytic Activity by Diffusion-Based and Tunneling Modes of Photo-Scanning Electrochemical Microscopy
Source: ACS Nano. 2025 Jan 10;19(3):3490–9. doi: 10.1021/acsnano.4c13276 (PMC11781031; doi:10.1021/acsnano.4c13276)
Supplement: Supplementary file 1 — nn4c13276_si_001.pdf [file nn4c13276_si_001.pdf]

Supporting Information for

High-Resolution Mapping of Photocatalytic Activity by Diffusion-Based and Tunneling Modes of Photo-Scanning Electrochemical Microscopy

Tianyu Bo,[†,§] *Debjit Ghoshal,*[‡] *Wilder, Logan,*[‡] *Elisa M. Miller,*[‡,]* *and Michael V. Mirkin*[†,%,]*

[†] Department of Chemistry and Biochemistry, Queens College, Flushing, NY 11367.

[§] The Graduate Center of CUNY, New York, NY 10016.

[‡] Materials, Chemistry, and Computational Science Directorate, National Renewable Energy Laboratory, Golden, CO 80401.

[%] Advanced Science Research Center at The Graduate Center, CUNY; New York, NY 10031.

e-mail: Elisa.Miller@nrel.gov, mmirkin@qc.cuny.edu

Table of contents:

Figure S1. Tunneling steady-state voltammograms of $Fe(CN)_6^{4-}$ at Pt and C tips touching a MoS_2 CVD-grown triangle.

Figure S2. Representative AFM image and line scan of a mixed-phase MoS_2 nanosheet.

Figure S3. Optical image of CVD grown MoS_2 triangles and Raman measurement of a monolayer triangle.

Figure S4. TEM images of representative Pt and C tips.

Figure S5. Raman spectra of the mixed-phase exfoliated MoS_2 on a Si substrate.

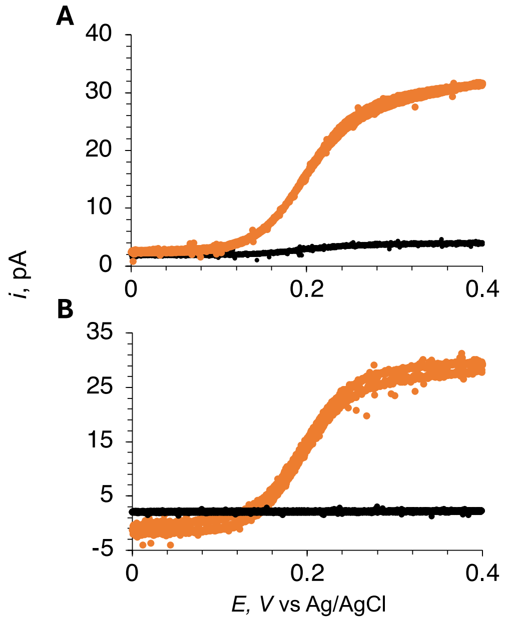

Figure S1. Tunneling steady-state voltammograms of $Fe(CN)_6^{4-}$ oxidation at (A) carbon tip and (B) Pt tip touching a MoS_2 CVD-grown triangle.

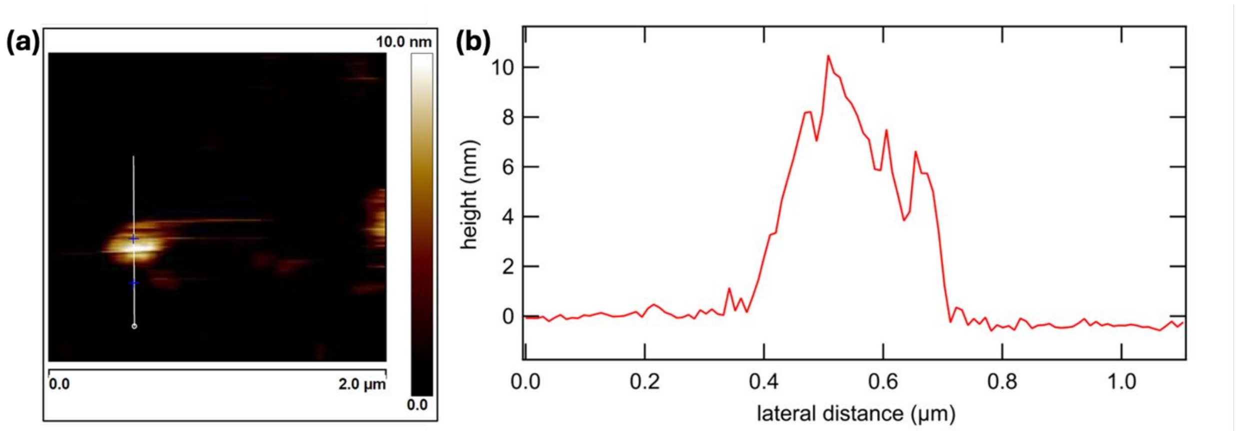

Figure S2. Representative AFM (a) image and (b) line scan of a mixed-phase MoS_2 nanosheet.

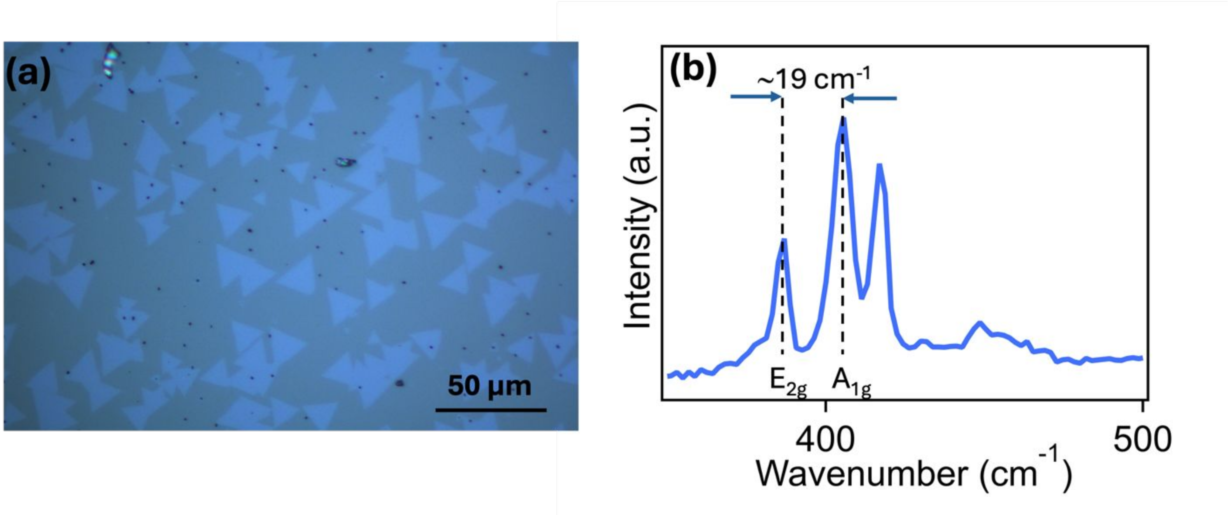

Figure S3. (a) Optical image of CVD grown MoS_2 triangles and (b) Raman measurement of a monolayer triangle.

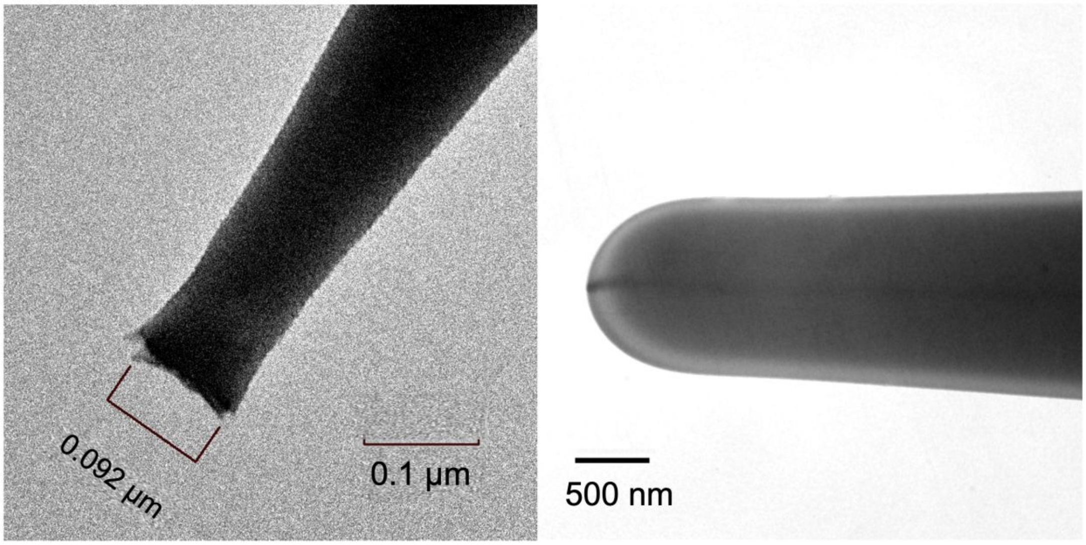

Figure S4. TEM images of representative (A) carbon and (B) microforged Pt tips.

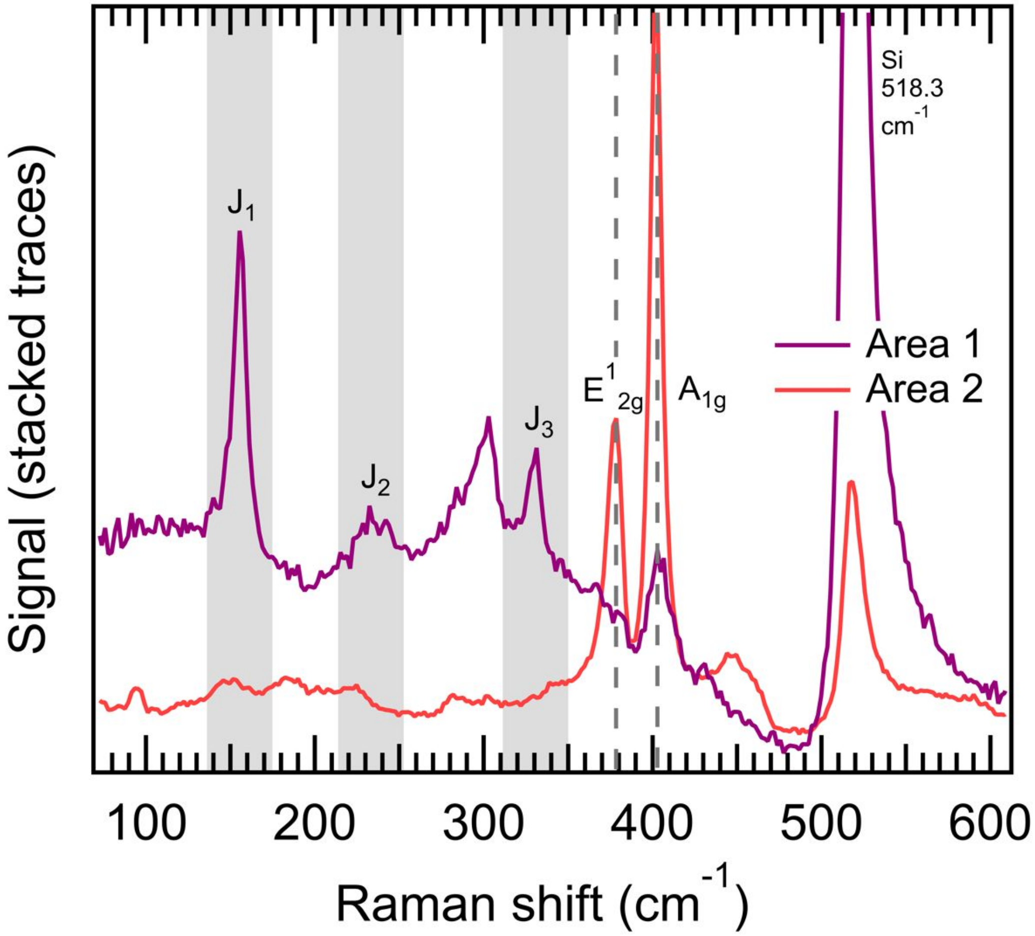

Figure S5. Raman spectra of solution-exfoliated MoS_2 flakes deposited on Si showing mixed 1T'/2H character, where area 1 and area 2 are measured from different flakes in the same drop cast sample. Note: The solution exfoliated MoS_2 flakes for Raman analysis were synthesized in a separate batch from the material used for electrochemical analysis but the same experimental procedure was followed.
